# A comparative network analysis to explore cancer patient experiences with cannabis

**DOI:** 10.3389/fpsyt.2026.1737119

**Published:** 2026-08-14

**Authors:** Apoorva Chada Reddy, Youning Zhao, Matthew Lowe, Zhiqiang Cai, Ryan Vandrey

**Affiliations:** 1 University of Wisconsin, Madison, WI, United States; 2 University of Pennsylvania, Philadelphia, PA, United States; 3 Realm of Caring Foundation, Colorado Springs, CO, United States; 4 Johns Hopkins University, Baltimore, MD, United States

**Keywords:** cancer, cannabis, CBD, epistemic network analysis, patient-reported outcomes, THC

## Abstract

**Introduction:**

Approximately 20% of cancer patients report cannabis use, yet only 30% of oncologists feel sufficiently informed to make recommendations on its use. This study aimed to visualize the network of themes that arise within cancer patients’ reported experiences with cannabis.

**Materials and methods:**

Data was collected via an online survey of 65 patients who self-reported the use of cannabis in their treatment for cancer, details about the cannabis product(s) being used, their perceived benefits and problems associated with cannabis use, their reasons for starting cannabis use, and any reasons for stopping cannabis use. Epistemic Network Analysis (ENA) was used to compare two groups of cancer patients: 1) those who only used CBD-dominant products (CBD-only group) versus 2) those who used cannabidiol (CBD)- and delta-9-tetrahydrocannabinol (THC)-containing products (either CBD-dominant and THC-dominant cannabis products or cannabis products containing a balanced ratio of both CBD and THC; CBD+THC group).

**Results:**

Cannabis use conferred therapeutic benefits for several health issues commonly encountered by cancer patients. Common benefits reported across the cohort of patients included pain relief, improved sleep, physical relaxation, emotional regulation, and reduction of concomitant medication. The most frequently reported barriers to cannabis use were the stigma associated with THC use and the high cost of CBD-dominant and THC-dominant products. Pain relief emerged as the most prominent, interconnected theme reported by the CBD+THC group, whereas emotional regulation was the most prominent theme for the CBD-only group.

**Conclusion:**

Symptom relief differed based on the cannabinoid composition of the cannabis products. The following trends emerged, which must be confirmed with larger samples: pain relief was more prominent in responses from users of CBD+THC, whereas emotional regulation was more prominent in only the users of CBD-only products. These findings are a step toward assisting cancer patients and providers with clinical decision-making on cannabis use. This study highlights the continued perception of stigma associated with THC use and the need for insurance coverage of medicinal cannabis to reduce the financial burden for this patient population. Finally, this study exemplifies the value of ENA in studying the therapeutic utility of cannabis with qualitative data.

## Highlights:

- Symptom relief for cancer patients differs based on the THC and CBD content of their cannabis products.
- Pain relief is more frequently reported among users of CBD+THC products.
- Emotional regulation is more frequently reported among users of only CBD.

## Introduction

Currently, approximately 1 in 5 cancer patients reports past-month cannabis use, often to manage adverse effects associated with cancer treatment (1, 2). There are three widely approved synthetic cannabinoid products available by prescription for cancer patients: Marinol (dronabinol; encapsulated delta-9-tetrahydrocannabinol (THC) in sesame oil), Syndros (dronabinol; THC in a liquid solution), and Cesamet (nabilone; encapsulated THC analog). These products are indicated for chemotherapy-induced nausea and vomiting in many jurisdictions (3). However, as the legalization of medicinal and non-medicinal (also called “recreational”) use of cannabis expands, cancer patients increasingly report using non-prescription cannabis products for therapeutic purposes, primarily to improve sleep and to relieve pain (4–6). Preclinical research and a few clinical studies suggest that two phytocannabinioids (chemical entities naturally occurring in the cannabis plant), cannabidiol (CBD) and THC, may help alleviate multiple symptoms associated with cancer and cancer treatment (e.g., pain, fatigue, anxiety, lack of appetite, nausea, and vomiting) and are likely safe with appropriate dosing (7–13). However, there is still much to be learned about cannabinoids before the purported health effects can be confirmed (14, 15).

Despite the increasing use of non-prescription cannabinoid products, cancer patients who seek these products may encounter several barriers. First, they may face a lack of clinical guidance. There are few randomized clinical trials on the effects of cannabis in cancer patients, and only 30% of oncologists feel sufficiently informed to make recommendations on cannabis use for their patients (14–16). Second, patients may face a high financial cost due to a lack of insurance coverage for cannabis products in many places that have legalized patient access (17). Third, there are social costs of cannabis use due to residual stigma from the anti-cannabis propaganda in the 1900s (18–20). Fourth, there are legal risks of cannabis use faced by patients who live where medicinal cannabis use remains prohibited or are subject to workplace drug testing, where THC detection could result in termination of employment (21). However, these barriers have not halted the cannabis legalization movement and the surging preference for non-prescription cannabinoids among cancer patients. With over two million new cancer cases predicted for the year 2025 in the United States alone, there is an urgent need for an understanding of cannabinoid safety and efficacy in cancer patients (22, 23). To inform the testing of cannabinoid products and the development of guidelines for efficacious cannabinoid use, it is important to uncover any patterns in the occurrence of positive and negative experiences associated with cannabis use among cancer patients.

New methods of analyzing patient-reported experiences with cannabis are becoming useful to understand the health impact of medicinal cannabis use. The purpose of this study was to visualize the network of themes that arise within cancer patient-reported descriptions of their experiences with cannabis. To visualize the connections among key themes, this study utilized Epistemic Network Analysis (ENA). ENA is a method for modeling and quantifying the connections in qualitative data, particularly through the co-occurrence of codes within conversations. It generates a weighted network of these connections and provides visualizations for each unit of analysis (24). By doing this, ENA allows researchers to interpret the themes more directly and mitigates the limitations of mere qualitative analysis by providing quantifiable connections. For this study, ENA was used to visualize themes within cancer patients’ open-ended responses on the benefits and problems of therapeutic cannabinoid use, reasons for starting, and reasons for stopping use.

## Materials and methods

Free-text survey data was collected from a sample of 65 adult cancer patients who reported cannabis use and completed at least one online survey as part of a larger study conducted by Johns Hopkins University School of Medicine and the Realm of Caring Foundation (RoC) (25). Participants were recruited through social media posts and word-of-mouth communication, and the health condition for which they were using cannabis as a therapeutic was self-reported (26). The dataset included cancer patients’ responses to the following questions: 1) “How has therapeutic use of cannabis/cannabinoids helped you?”; 2) “How has therapeutic use of cannabis/cannabinoids harmed or caused problems for you?”; 3) “Why did you choose to begin therapeutic use of cannabinoids?”; and 4) “[If they stopped], why did you stop medical use of cannabis/cannabinoids?” This approach helped visualize recurring themes and their interconnections, offering a clear picture of participants’ experiences with therapeutic cannabis use. The dataset also included information on the specific health condition for which the patient was trying to treat with cannabis, the type of cannabis product(s) (THC, CBD, or a combination of THC and CBD) used by participants, and basic participant demographics (26).

The codebook used in this study was derived from Leventhal’s Common-Sense Model of Self-Regulation (CSM). According to CSM, when an individual (e.g., cancer patient) notices their self-appraisal differs from expected due to a new or worsening symptom, the individual generates an emotional response and mental representation of their condition, informed by their socio-cultural context (27, 28). Individuals cope by iterating through a series of interventions (e.g., cannabis use) and self-evaluations to self-regulate (29). CSM guided the development of a codebook that reflects aspects of cancer patient experiences with cannabis supported by existing literature, as shown in **Table 1** (13, 16–18, 30). The final codebook included thematic codes, definitions, and examples. Two human coders independently coded a random subset of 30 responses, compared coding decisions, discussed discrepancies, and refined code definitions before applying the finalized codebook to the full dataset. Coding congruence was assessed using Cohen’s kappa (**Table 1**). No artificial intelligence tools were used to code the data or corroborate coding decisions.

**Table 1:** Codebook.

Theme	Definition	Example	Kappa
Pain Relief	Cannabis use alleviated pain.	*“Helps with severe pain”*	0.99
Emotional Regulation	Cannabis use relieved negative emotions and improved positive emotions and life outlook.	*“CBD oil helps with sense of overall well-being.”*	1.00
Sleep or Physical Relaxation	Cannabis use relaxed the body and improved sleep quality.	*“Helps me to sleep”*	1.00
Appetite Stimulation	Cannabis use reduced nausea or increased appetite.	*“Definitely reduced nausea.”*	1.00
Condition Improvement	Cannabis use treated a disease or condition.	*“Help fight cancer and to keep me healthy.”*	0.99
Medication Reduction	Cannabis use mitigated side effects and reduced the need for other medications.	*“I’m able to take a much lower dose of pharmaceuticals.”*	1.00
Lack of Guidance	Uncertainty about how to use cannabis due to lack of professional medical advice.	*“Not sure of proper regimen to do.”*	0.92
No Effect or Adverse Effect	Cannabis provided no benefit or caused negative effects.	*“Makes me feel slower than usual.”*	0.97
Stigma	Perceived social, professional, or legal barriers to cannabis use.	*“Negative relationships with in-laws and friends.”*	1.00
High Cost	The financial cost of cannabis was a barrier to use.	*“Cannot Afford it with my Disability Income.”*	1.00

### Analytic approach

Epistemic Network Analysis (ENA) is a method for modeling the structure of connections among codes in qualitative data by quantifying code co-occurrences within defined conversations. In this study, ENA began after human coding was completed and used the codes assigned to each response to construct weighted networks.

Responses were categorized into two cannabinoid-exposure categories based on the product type reported with each response: CBD-only and CBD+THC. The “CBD-only” group refers to responses associated with reported use of products containing cannabidiol (CBD) as the primary chemical constituent. The “CBD+THC” group refers to responses associated with reported use of both CBD-dominant and THC-dominant cannabis products or products that contained a balanced ratio of both CBD and THC.

In the ENA model, cannabinoid exposure category and user ID were defined as unit variables. Individual survey responses were treated as the conversation level, with a moving stanza size of 1, so code co-occurrences were calculated only within the same response line. This specification was used because some participants reported different exposure statuses across responses, and it prevented co-occurrences from being calculated across statements reflecting different exposure categories. Only non-missing responses contributed to the network corresponding to that survey question. Participants were therefore retained in the analysis even if they did not answer every open-ended question.

A comparative approach was used to characterize the distinctions between CBD-only and CBD+THC exposure groups. The comparative analysis involved calculating the absolute difference in line weights between the CBD and CBD+THC exposure ENA models. Line weights correspond to the strength of the relationship between each pair of themes coded in this study and depict the frequency of co-occurrence of codes in responses from each cancer patient (31). A two-sample t-test was used to test for differences between the salient themes in survey responses from cancer patients in the CBD+THC group versus CBD-only group. Given the self-selected nature of the sample, the t-test was exploratory and intended only to support the comparative visualization generated through ENA.

## Results

The average age of the sample was 53 years. 75% of the sample was White. All participants used a CBD-dominant cannabis product, and approximately half (49%) of the sample also used a THC-dominant product or a product that contained roughly equal amounts of CBD and THC. 92% of the sample were currently using cannabis at the time of survey completion (some participants had stopped prior use at the time follow-up assessments were completed (**Table 2**). The most common type of cannabis product used was CBD-dominant cannabis oil (**Table 3**). Pain relief was the most frequently coded theme in patient-reported descriptions of cannabis product effects (**Table 4**).

**Table 2:** Sample characteristics of cancer patients (N = 65).

Characteristic	N	%
Age		
18-50	25	39
51-100	40	61
Sex		
Female	49	75
Male	16	25
Race and ethnicity		
White	49	75
Multi-racial	5	7
Hispanic or Latino	3	5
Other/Unknown	8	12
Cannabinoid Exposure		
CBD	33	51
CBD+THC	32	49
Current cannabis use		
Yes	60	92
No	5	8

**Table 3:** Frequency of cannabis product form and cannabinoid content.

Cannabis product	Number of patients using	%
Cannabis oil CBD-dominant	19	29
Cannabis oil THC-dominant	11	17
Cannabis edibles THC-dominant	11	17
Cannabis flowers unknown THC/CBD content	10	15
Cannabis oil unknown THC/CBD content	9	14
Cannabis flowers THC-dominant	8	12
CBD patch/gel	8	12
Cannabis flowers CBD-dominant	7	11
Cannabis edibles CBD-dominant	6	9
Cannabis flowers balanced THC/CBD	5	8
Cannabis edibles unknown THC/CBD content	5	8
Cannabis concentrates	3	5
Dronabinol	1	2
Other cannabinoid product	7	11

This table shows the patient-reported cannabis product form and content.

**Table 4:** Frequency of codes.

Code	Number of instances	%
Pain Relief	112	21
Emotional regulation	81	15
Medication Reduction	75	14
Sleep or Physical Relaxation	52	10
Condition Improvement	44	8
No Effect or Adverse Effect	40	8
Stigma	24	5
Appetite Stimulation	22	4
High Cost	20	4
Lack of Guidance	5	1

This table shows the number of times a particular code appeared in the participant responses.

The frequency of each of the cannabis product use types in the dataset is shown in **Table 3**.

The frequency of each code in the dataset is shown in **Table 4**.

A two-sample t-test supported categorizing participants into two groups (CBD-only vs. CBD+THC). The test showed a significant difference overall in the effects of cannabis described by the CBD-only and CBD+THC groups (p < 0.01). The effect size, measured by Cohen’s d, was 0.75 (95% CI: 0.29-1.20), indicating a large effect and confirming a meaningful difference in the content of responses between the two groups.

**Figure 1:**
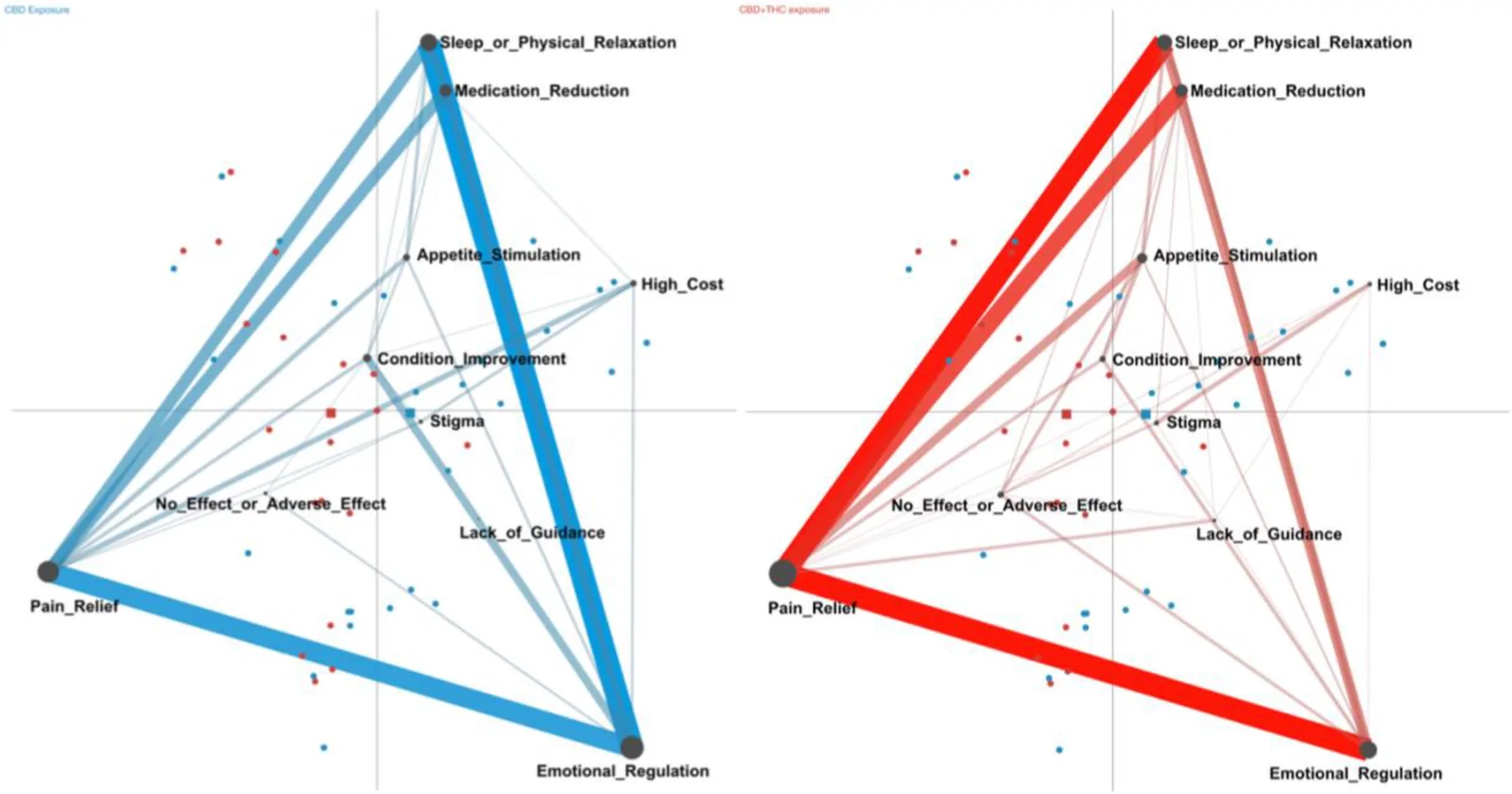
ENA models for CBD exposure (blue) and CBD+THC exposure (red). Nodes represent coded themes, and lines represent code co-occurrences within response lines; thicker lines indicate stronger co-occurrence. Each dot represents a unit defined by user ID within cannabinoid exposure category. Node positions are fixed across exposure categories to enable visual comparison of within-category network patterns. The axes represent the primary dimensions of variance in the ENA projection.

Building on this foundation, ENA was used to explore common themes shared by both groups, defined by cannabinoid exposure as CBD-only (shown in blue) and CBD+THC (shown in red) (**Figure 1**). The ENA analysis model demonstrated excellent goodness of fit, achieving Pearson correlation coefficients of 0.88 and 0.93 for the X and Y axes, respectively. This indicated a robust model suitable for analysis.

As shown in **Figure 1**, emotional regulation had the strongest connections in the CBD-only group, and pain relief had the strongest connections in the CBD+THC group. Pain relief, emotional regulation, sleep or physical relaxation, and medication reduction emerged as benefits experienced by cancer patients in both CBD-only and CBD+THC exposure groups.

Pain relief was the most strongly connected node in the CBD+THC group and the second-most strongly connected node in the CBD-only group, indicating it is a frequently discussed theme in patients’ responses. The themes linked to pain relief were largely consistent across both groups, though their relative strengths differed. For the CBD-only group (blue), pain relief was most strongly associated with emotional regulation (line weight = 0.16), medication reduction (line weight = 0.09), sleep or physical relaxation (line weight = 0.09), and appetite stimulation (line weight = 0.04). In comparison, for the CBD+THC group (red), pain relief exhibited strong connections with sleep or physical relaxation (line weight = 0.18), emotional regulation (line weight = 0.18), medication reduction (line weight = 0.14), and appetite stimulation (line weight = 0.07). **Table 5**, Row 1 shows quotes that exemplify the pain relief code.

**Table 5:** Patient quotes.

Row	Code(s)	Example quotes
1	Pain Relief	*“(It helped with) sleep issues and body pain.” (CBD+THC group)* *“It allows me to escape chronic pain and helps with giving me an appetite. I would not be able to sleep at all if it weren’t for it.” (CBD+THC group)*
2	Emotional Regulation	*“CBD oil helps with constipation, digestive issues, sense of overall well being, immune system, feel better.” (CBD-only group)* *“(It helps with) sleep, mood, pain.” (CBD+THC group)*
3	Sleep or Physical Relaxation	*“Has increased my ability to eat, sleep, and life quality.” (CBD-only group)* *“(Helped) with sleep; my depression and anxiety; helps me to be focused; helps bring pain level down where I can ‘deal with it better’….I will NEVER be without it!” (CBD+THC group)*
4	Medication Reduction	*“Better pain killer than any of my prescription drugs or OTC or herbs, etc.” (CBD-only group)* *“(I started since) the prescriptions side effects are awful and I’m still suffering with pain on them. (but with cannabis) I’ve been able to lower my meds.” (CBD+THC group)*
5	High Cost	*“Unable to access it in this location; seems to help with anxiety, pain, inflammation. “ (CBD-only group)* *“Elderly (family) members in fear with (conflicting) belief systems have suffered shock upon hearing that it could even be possible that I was using cannabis to manage my health issues. Also the cost.” (CBD+THC group)*
6	Emotional Regulation + Sleep or Physical Relaxation	*“Helps me relax and helps with the severe anxiety from the severe chronic pain no doubt.” (CBD-only group)* *“Helps with sleep slightly, constipation, sense of well-being.” (CBD-only group)*
7	High Cost + Pain Relief	*“The Hemp Oil helps with spasms in my foot and leg. The THC: CBD tincture when I can afford it helps with pain.” (CBD-only group)* *“Lack of income to purchase charlottes web. I wish I could afford it right now because after a week of taking it my mood got better and my pain started to go away, my blood pressure went down and my diabetes went away. at 10ml once daily” (CBD-only group)*
8	Lack of Guidance	*“The two biggest problems that I have is that I feel that I’m unable to provide myself with the necessary dose to really attack cancer (because) I get (too) out of it and, I’m allergic to the point of waking with sinus issues and headache that usually dissipate by afternoon.” (CBD+THC group)* *“I have read studies that have shown apoptosis of cancer cells. However studies also show that it may be necessary to take 1000 mg a day. I don’t know if my syringe equals a total of 200mg. Or total 400mg. But I’m taking half a syringe a day.” (CBD+THC group)*

This table shows quotes from patient survey responses exemplifying common ENA code combinations.

Emotional regulation had partially overlapped, but differently weighted associations in each cannabinoid exposure group (**Table 5**, Row 2). In the CBD-only group (blue), emotional regulation was most associated with sleep or physical relaxation (line weight = 0.18), pain relief (line weight = 0.16), medication reduction (line weight = 0.06), and condition improvement (line weight = 0.06). For the CBD+THC group (red), emotional regulation was most associated with pain relief (line weight = 0.18), followed by sleep or physical relaxation (line weight = 0.08), condition improvement (line weight = 0.03), and medication reduction (line weight = 0.03).

Across both groups, the benefits that frequently co-occurred with sleep or physical relaxation were medication reduction (line weight = 0.06 for CBD-only, 0.04 for CBD+THC), pain relief (line weight = 0.09 for CBD-only, 0.18 for CBD+THC), appetite stimulation (line weight = 0.02 for CBD, 0.03 for CBD+THC), and emotional regulation (line weight = 0.18 for CBD-only, 0.08 for CBD+THC). This is exemplified in **Table 5**, Row 3.

Medication reduction was the most frequently associated with pain relief (line weight = 0.09 for CBD-only, 0.14 for CBD+THC). This is exemplified in **Table 5**, Row 4.

High cost and stigma emerged as barriers to cannabinoid use. High cost was most strongly associated with pain relief in the CBD-only group (line weight = 0.04) and with stigma in the CBD+THC group (line weight = 0.04). Participants often highlighted the burden of cost in relation to accessing treatment (**Table 5**, Row 5).

**Figure 2:**
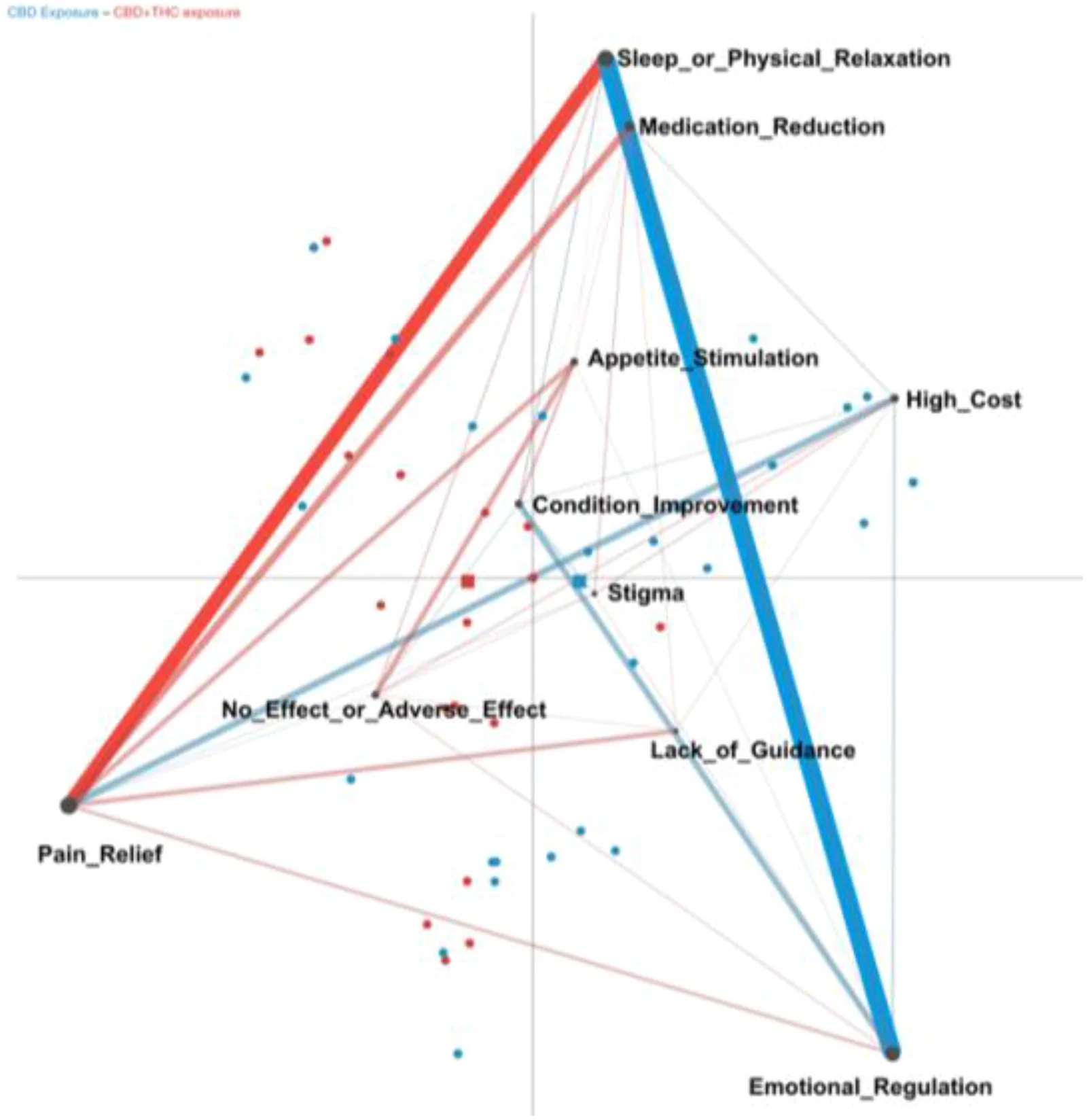
ENA comparison model between CBD-only (blue) and CBD+THC (red). This figure shows the difference network comparing connection strengths between the two exposure categories. Blue lines indicate connections that were stronger in the CBD-only network, and red lines indicate connections that were stronger in the CBD+THC network. Thicker lines indicate larger differences in connection strength. Nodes remain in the same positions as in **Figure 1** to support direct comparison.

**Figure 2** visualizes thematic differences between responses categorized as CBD-only or CBD+THC. The difference in the connection between emotional regulation and sleep or physical relaxation across cannabinoid groups did not reach statistical significance (p = 0.067; CBD-only line weight = 0.18; CBD+THC line weight = 0.08). This is exemplified in **Table 5**, Row 6. Therefore, this result was not interpreted as evidence of a group difference, though the observed pattern may be examined in future studies with larger samples.

Similarly, the difference in connection between high cost and pain relief did not reach statistical significance across cannabinoid groups (p = 0.08; CBD-only line weight = 0.04; CBD+THC line weight = 0.005). This is exemplified in **Table 5**, Row 7. This result was also interpreted descriptively rather than as evidence of a group difference.

In this dataset, perceived lack of guidance was reported only in responses categorized as CBD+THC exposure. Within the CBD+THC group, lack of guidance was loosely connected to the following themes: no effects or adverse effects, high cost, medication reduction, and pain relief (line weights < 0.01). Adverse effects reported by the CBD-only group included: bad dreams and an upset stomach after initial CBD use and after using too much CBD. Adverse effects reported by the CBD+THC group included: daytime sleepiness, lethargy, feeling high, paranoia, anxiety, and increased appetite leading to weight gain (**Table 5**, Row 8).

## Discussion

This study confirms and extends existing literature through a visual depiction of the interconnected nature of the perceived benefits and challenges of therapeutic cannabinoid use among cancer patients. Consistent with recent studies, pain relief was the most frequently discussed benefit among all cancer patients in this sample (32). Pain relief was the most prominent benefit experienced by the CBD+THC group, supporting previous studies that found THC and 1:1 CBD+THC products to be efficacious for pain relief (33). Pain relief was the second-most prominent benefit in the CBD-only group, an important finding given the mixed evidence on whether CBD provides benefits over placebo for pain (34). The differences in findings on CBD’s effect on pain may, in part, be explained by differences in methods of measuring pain (34). Statistical inferences should be interpreted with caution due to the non-random nature of the sample.

Across both CBD and CBD+THC groups, pain relief was closely linked with improved sleep, emotional regulation, and medication reduction, and moderately linked with appetite stimulation. This finding aligns with recent surveys reporting that sleep, anxiety, pain, and appetite are among the top reasons cancer patients reported using cannabis (6). Cancer patients have also reported reducing their opioid medication intake by using cannabis for symptom relief (35). Although cannabis is not classified as an anti-cancer agent, its promise in palliative care as an adjuvant therapy is evident (35, 36).

In the CBD+THC group, the strongest association was between pain relief and enhancements in sleep and physical relaxation. This aligns with pharmacological research that attributes sedative properties to acute use of THC (37, 38). In contrast, this pain-sleep link was weaker in the CBD-only group. The impact of CBD on sleep tends to be more variable (39–42).

Emotional regulation was the most prominent theme among CBD-only users. It is possible that by reducing other medications they were dependent on for pain relief, and improving emotional regulation, their ability to manage pain improved. Studies suggest opioid use prolongs neuropathic pain (43).

In the CBD-only group, the strongest associations were among emotional regulation, sleep, and physical relaxation. This affirms existing literature showing that CBD reduces anxiety, improves mood, and promotes relaxation, possibly by modulating serotonin and anxiety-regulating pathways, including the hypothalamic-pituitary-adrenal axis (44, 45). While differences between exposure groups in the emotional regulation–sleep/relaxation connection did not reach statistical significance, the numerical pattern may warrant further examination in larger samples. Research is also needed to confirm the mechanisms underlying these effects.

Stigma and financial burden were common barriers in both groups. Qualitative studies have similarly identified stigma and financial burden as significant barriers to cannabis use among cancer patients (46).

High cost was more strongly tied to pain relief in the CBD-only group, potentially reflecting higher dose requirements for comparable relief. In previous studies, patients using CBD reported that cost was a limiting factor in continued use, with some ceasing cannabis therapy due to financial constraints (46). Cannabis is currently an out-of-pocket expense, creating disparities in access (47, 48). The greater strength of the association between high cost and pain relief in the CBD-only group may be attributed to higher CBD dosage recommendations compared with THC-dominant products. Despite limited clinical evidence to determine dosing for cancer-related pain relief (49), guidelines for patients without prior cannabis use have been proposed in which 10 mg of CBD is taken per day and increased up to 40 mg if needed (50, 51). For experienced cannabis users, the guidelines recommend starting at a combination of 2.5 mg of CBD and 2.5 mg of THC per day (50, 51). Thus, the higher dosage recommendation for CBD-only use may explain the higher perceived financial burden among these patients. Note, however, that these guidelines were developed for treating chronic pain and may not extend to cancer or other health conditions for which cannabis is being considered for therapeutic purposes. Alternatively, the association between high cost and pain relief may be attributable to a cost-expectancy effect (i.e., the more a patient pays, the more likely they are to rate it as beneficial) (52).

Only the CBD+THC group expressed concerns about a lack of guidance. This makes sense, considering the stigma surrounding THC use and the clinical guidelines stating that THC should not be used for the very symptoms that are often medicated with THC by cancer patients. For example, despite pain being a common reason for CBD+THC use among cancer patients, the Multinational Association for Supportive Care in Cancer recently recommended against the use of cannabis for cancer-related pain due to insufficient supporting scientific evidence (53). Given the prevalence of cannabis use among cancer patients, this suggests that a substantial proportion of patients are using cannabis outside of formal medical supervision.

Self-selection bias and self-report bias are limitations of this study; self-reported product types without laboratory verification introduce uncertainty into the cannabinoid-type groupings. Additionally, multiple network connections and line weights are compared without adjustment, increasing the risk of Type I error. The lack of diversity in the sample also limits the generalizability of these results to more diverse populations. Future research with larger, more diverse samples should incorporate objective measures of cannabinoid efficacy and investigate long-term outcomes. Additionally, exploring the differential effects of specific cannabinoid formulations could refine therapeutic approaches. Future research should compare current versus former cannabis users and should ideally include a non-cannabis-using control group with a larger sample in a randomized trial. Cancer symptoms vary in type and severity depending on the cancer type and stage, as well as on treatment, neither of which was collected in this study (54, 55). Future research should compare 1) patients with different cancer types and 2) patients with different cancer staging and treatment to identify differences in the perceived effects of different cannabinoid content and routes of administration.

Overall, this study provides new insights by identifying distinct networks of co-occurring symptoms and perceived benefits that vary with cannabinoid composition. This study provides both a visual and quantitative comparison of the effects of CBD+THC and CBD products. Overall, pain relief was the most frequently discussed benefit and was associated with other benefits: emotional regulation, sleep or physical relaxation, and medication reduction. Primary patient concerns were stigma and cost. The order of importance of benefits and concerns differed based on cannabinoid exposure. These findings provide a framework for designing future studies that evaluate symptom clusters rather than isolated endpoints.

## Data Availability

The raw data supporting the conclusions of this article will be made available by the authors, without undue reservation.

## References

[1] Pergam SA Woodfield MC Lee CM Cheng GS Baker KK Marquis SR . Cannabis use among patients at a comprehensive cancer center in a state with legalized medicinal and recreational use. Cancer. (2017) 123:4488–97. doi: 10.1002/cncr.30879 28944449 PMC5698756

[2] Kim A Kaufmann CN Ko R Li Z Han BH . Patterns of medical cannabis use among cancer patients from a medical cannabis dispensary in New York state. J Palliative Med. (2019) 22:1196–201. doi: 10.1089/jpm.2018.0529 30909786 PMC6776253

[3] U. S. Food and Drug Administration . FDA and Cannabis: Research and Drug Approval Process (2023). Available online at: https://www.fda.gov/news-events/public-health-focus/fda-and-cannabis-research-and-drug-approval-process (Accessed July 3, 2026).

[4] Blake EA Ross M Ihenacho U Figueroa L Silverstein E Flink D . Non-prescription cannabis use for symptom management amongst women with gynecologic Malignancies. Gynecol Oncol Rep. (2019) 30:100497. doi: 10.1016/j.gore.2019.100497 31692541 PMC6804884

[5] Brasky TM Newton AM Conroy S Adib A Adley NC Strassels SA . Marijuana and cannabidiol use prevalence and symptom management among patients with cancer. Cancer Res Commun. (2023) 3:1917–26. doi: 10.1158/2767-9764.crc-23-0233 37772996 PMC10515742

[6] Azizoddin DR Cohn AM Ulahannan SV Henson CE Alexander AC Moore KN . Cannabis use among adults undergoing cancer treatment. Cancer. (2023) 129:3498–508. doi: 10.1002/cncr.34922 37354093 PMC11070130

[7] Kleckner AS Kleckner IR Kamen CS Tejani MA Janelsins MC Morrow GR . Opportunities for cannabis in supportive care in cancer. Ther Adv Med Oncol. (2019) 11:1758835919866362. doi: 10.1177/1758835919866362 31413731 PMC6676264

[8] Farrell C Brearley SG Pilling M Molassiotis A . The impact of chemotherapy-related nausea on patients' nutritional status, psychological distress and quality of life. Support Care Cancer. (2013) 21:59–66. doi: 10.1007/s00520-012-1493-9 22610269

[9] Madeo G Kapoor A Giorgetti R Busardò FP Carlier J . Update on cannabidiol clinical toxicity and adverse effects: A systematic review. Curr Neuropharmacol. (2023) 21:2323–42. doi: 10.2174/1570159x21666230322143401 36946485 PMC10556379

[10] Arnold JC . A primer on medicinal cannabis safety and potential adverse effects. Aust J Gen Pract. (2021) 50:345–50. doi: 10.31128/ajgp-02-21-5845 34059837

[11] National Academies of Sciences E Medicine . The Health Effects of Cannabis and Cannabinoids: The Current State of Evidence and Recommendations for Research. Washington, DC: The National Academies Press (2017). 28182367

[12] Zagzoog A Mohamed KA Kim HJJ Kim ED Frank CS Black T . *In vitro* and *in vivo* pharmacological activity of minor cannabinoids isolated from Cannabis sativa. Sci Rep. (2020) 10:20405. doi: 10.1038/s41598-020-77175-y 33230154 PMC7684313

[13] Abrams DI . The therapeutic effects of cannabis and cannabinoids: an update from the National Academies of Sciences, Engineering and Medicine report. Eur J Internal Med. (2018) 49:7–11. doi: 10.1016/j.ejim.2018.01.003 29325791

[14] Steele G Arneson T Zylla D . A comprehensive review of cannabis in patients with cancer: Availability in the USA, general efficacy, and safety. Curr Oncol Rep. (2019) 21:10. doi: 10.1007/s11912-019-0757-7 30707319

[15] Crichton M Dissanayaka T Marx W Gamage E Travica N Bowers A . Does medicinal cannabis affect depression, anxiety, and stress in people with cancer? A systematic review and meta-analysis of intervention studies. Maturitas. (2024) 184:107941. doi: 10.1016/j.maturitas.2024.107941 38430618

[16] Braun IM Wright A Peteet J Meyer FL Yuppa DP Bolcic-Jankovic D . Medical oncologists’ beliefs, practices, and knowledge regarding marijuana used therapeutically: a nationally representative survey study. J Clin Oncol. (2018) 36:1957. doi: 10.1200/jco.2017.76.1221 29746226 PMC6553839

[17] Philbrick AM . Up in smoke: One state's perspective on the affordability of medical cannabis. J Am Pharm Assoc (2003). (2020) 60:216–7. doi: 10.1016/j.japh.2019.09.008 31740295

[18] Valencia CI Asaolu IO Ehiri JE Rosales C . Structural barriers in access to medical marijuana in the USA-a systematic review protocol. Syst Rev. (2017) 6:154. doi: 10.1186/s13643-017-0541-4 28784163 PMC5547531

[19] Carliner H Brown QL Sarvet AL Hasin DS . Cannabis use, attitudes, and legal status in the U.S.: A review. Prev Med. (2017) 104:13–23. doi: 10.1016/j.ypmed.2017.07.008 28705601 PMC6348863

[20] Solomon R . Racism and its effect on cannabis research. Cannabis Cannabinoid Res. (2020) 5:2–5. doi: 10.1089/can.2019.0063 32322671 PMC7173675

[21] Hazle MC Hill KP Westreich LM . Workplace cannabis policies: A moving target. Cannabis Cannabinoid Res. (2022) 7:16–23. doi: 10.1089/can.2020.0095 33998870 PMC8864412

[22] Siegel RL Kratzer TB Giaquinto AN Sung H Jemal A . Cancer statistics, 2025. CA Cancer J Clin. (2025) 75:10–45. doi: 10.3322/caac.21871 39817679 PMC11745215

[23] State medical cannabis laws. In: National Conference of State Leglislatures. Denver, CO: National Conference of State Legislatures.

[24] Shaffer DW . Quantitative Ethnography, Lulu. Com. Madison, WI: Cathcart Press (2017).

[25] Schlienz NJ Scalsky R Martin EL Jackson H Munson J Strickland JC . A cross-sectional and prospective comparison of medicinal cannabis users and controls on self-reported health. Cannabis Cannabinoid Res. (2021) 6:548–58. doi: 10.1089/can.2019.0096 33998852 PMC8713273

[26] Martin EL Strickland JC Schlienz NJ Munson J Jackson H Bonn-Miller MO . Antidepressant and anxiolytic effects of medicinal cannabis use in an observational trial. Front Psychiatry. (2021) 12:729800. doi: 10.3389/fpsyt.2021.729800 34566726 PMC8458732

[27] Leventhal H Phillips LA Burns E . The common-sense model of self-regulation (CSM): a dynamic framework for understanding illness self-management. J Behav Med. (2016) 39:935–46. doi: 10.1007/s10865-016-9782-2 27515801

[28] Durazo A Cameron LD . Representations of cancer recurrence risk, recurrence worry, and health-protective behaviours: an elaborated, systematic review. Health Psychol Rev. (2019) 13:447–76. doi: 10.1080/17437199.2019.1618725 31117924 PMC6851428

[29] Cameron LD Leventhal H . The Self-Regulation of Health and Illness Behaviour. , In: Citeseer. London: Routledge (2003).

[30] Kvamme SL Pedersen MM Rømer Thomsen K Thylstrup B . Exploring the use of cannabis as a substitute for prescription drugs in a convenience sample. Harm Reduct J. (2021) 18:72. doi: 10.1186/s12954-021-00520-5 34246279 PMC8272272

[31] Hevey D . Network analysis: a brief overview and tutorial. Health Psychol Behav Med. (2018) 6:301–28. doi: 10.1080/21642850.2018.1521283 34040834 PMC8114409

[32] Gorzo A Havași A Spînu Ș Oprea A Burz C Sur D . Practical considerations for the use of cannabis in cancer pain management-what a medical oncologist should know. J Clin Med. (2022) 11(17):5036. doi: 10.3390/jcm11175036 36078963 PMC9457511

[33] McDonagh MS Morasco BJ Wagner J Ahmed AY Fu R Kansagara D . Cannabis-based products for chronic pain: a systematic review. Ann Intern Med. (2022) 175:1143–53. doi: 10.7326/m21-4520 35667066

[34] Mohammed SYM Leis K Mercado RE Castillo MMS Miranda KJ Carandang RR . Effectiveness of cannabidiol to manage chronic pain: A systematic review. Pain Manag Nurs. (2024) 25:e76–86. doi: 10.1016/j.pmn.2023.10.002 37953193

[35] Pawasarat IM Schultz EM Frisby JC Mehta S Angelo MA Hardy SS . The efficacy of medical marijuana in the treatment of cancer-related pain. J Palliat Med. (2020) 23:809–16. doi: 10.1089/jpm.2019.0374 32101075

[36] Shalata W Abu Saleh O Tourkey L Shalata S Neime AE Abu Juma’a A . The efficacy of cannabis in oncology patient care and its anti-tumor effects. Cancers (Basel). (2024) 16(16):2909. doi: 10.3390/cancers16162909 39199679 PMC11352579

[37] Velzeboer R Malas A Boerkoel P Cullen K Hawkins M Roesler J . Cannabis dosing and administration for sleep: a systematic review. Sleep. (2022) 45(11):zsac218. doi: 10.1093/sleep/zsac218 36107800

[38] Gorelick DA Goodwin RS Schwilke E Liu F Ortemann-Renon C Bonnet D . Tolerance to effects of high-dose oral δ9-tetrahydrocannabinol and plasma cannabinoid concentrations in male daily cannabis smokers. J Anal Toxicol. (2013) 37:11–6. doi: 10.1093/jat/bks081 23074216 PMC3584989

[39] Villanueva MRB Joshaghani N Villa N Badla O Goit R Saddik SE . Efficacy, safety, and regulation of cannabidiol on chronic pain: A systematic review. Cureus. (2022) 14:e26913. doi: 10.7759/cureus.26913 35860716 PMC9288157

[40] Vaillancourt R Gallagher S Cameron JD Dhalla R . Cannabis use in patients with insomnia and sleep disorders: retrospective chart review. Can Pharm J (Ott). (2022) 155:175–80. doi: 10.1177/17151635221089617 35519083 PMC9067069

[41] Kaul M Zee PC Sahni AS . Effects of cannabinoids on sleep and their therapeutic potential for sleep disorders. Neurotherapeutics. (2021) 18:217–27. doi: 10.1007/s13311-021-01013-w 33580483 PMC8116407

[42] Kaufmann R Bozer AH Jotte AK Aqua K . Long-term, self-dosing CBD users: Indications, dosage, and self-perceptions on general health/symptoms and drug use. Med Cannabis Cannabinoids. (2023) 6:77–88. doi: 10.1159/000531666 37900894 PMC10601936

[43] Grace PM Strand KA Galer EL Urban DJ Wang X Baratta MV . Morphine paradoxically prolongs neuropathic pain in rats by amplifying spinal NLRP3 inflammasome activation. Proc Natl Acad Sci. (2016) 113:E3441–50. doi: 10.1073/pnas.1602070113 27247388 PMC4914184

[44] Bloomfield MAP Yamamori Y Hindocha C Jones APM Yim JLL Walker HR . The acute effects of cannabidiol on emotional processing and anxiety: a neurocognitive imaging study. Psychopharmacol (Berl). (2022) 239:1539–49. doi: 10.1007/s00213-022-06070-3 35445839 PMC9110481

[45] Jenkins BW Spina HA Nicholson K Newman AEM Khokhar JY . Cannabidiol (CBD) potentiates physiological and behavioral markers of hypothalamic-pituitary-adrenal (HPA) axis responsivity in female and male mice. Psychopharmacol (Berl). (2026) 243:287–29. doi: 10.1007/s00213-024-06737-z 39754667

[46] Morris JN Loyer J Blunt J . Stigma, risks, and benefits of medicinal cannabis use among Australians with cancer. Support Care Cancer. (2024) 32:252. doi: 10.1007/s00520-024-08439-w 38532234

[47] Lapen K Mishra Meza A Dee EC Mao JJ Raghunathan NJ Jinna S . Patient out-of-pocket costs for cannabis use during cancer treatment. J Natl Cancer Inst Monogr. (2024) 2024:305–12. doi: 10.1093/jncimonographs/lgad030 39108238 PMC11303855

[48] Olson RE Smith A Good P Dudley M Gurgenci T Hardy J . 'What price do you put on your health?': Medical cannabis, financial toxicity and patient perspectives on medication access in advanced cancer. Health Expect. (2023) 26:160–71. doi: 10.1111/hex.13642 36335552 PMC9854313

[49] Braun IM Bohlke K Abrams DI Anderson H Balneaves LG Bar-Sela G . Cannabis and cannabinoids in adults with cancer: ASCO guideline. J Clin Oncol. (2024) 42:1575–93. doi: 10.1200/jco.23.02596 38478773 PMC11730458

[50] Bhaskar A Bell A Boivin M Briques W Brown M Clarke H . Consensus recommendations on dosing and administration of medical cannabis to treat chronic pain: results of a modified Delphi process. J Cannabis Res. (2021) 3:22. doi: 10.1186/s42238-021-00073-1 34215346 PMC8252988

[51] Jugl S Goodin AJ Brown JD . Climbing the evidence pyramid: Dosing considerations for medical cannabis in the management of chronic pain. Med Cannabis Cannabinoids. (2023) 6:41–5. doi: 10.1159/000530251 37124080 PMC10134049

[52] Waber RL Shiv B Carmon Z Ariely D . Commercial features of placebo and therapeutic efficacy. JAMA. (2008) 299:1016–7. doi: 10.1001/jama.299.9.1016 18319411

[53] To J Davis M Sbrana A Alderman B Hui D Mukhopadhyay S . MASCC guideline: Cannabis for cancer-related pain and risk of harms and adverse events. Support Care Cancer. (2023) 31:202. doi: 10.1007/s00520-023-07662-1 36872397

[54] Koo MM Swann R McPhail S Abel GA Elliss-Brookes L Rubin GP . Presenting symptoms of cancer and stage at diagnosis: Evidence from a cross-sectional, population-based study. Lancet Oncol. (2020) 21:73–9. doi: 10.1016/s1470-2045(19)30595-9 31704137 PMC6941215

[55] Deshields TL Potter P Olsen S Liu J . The persistence of symptom burden: Symptom experience and quality of life of cancer patients across one year. Support Care Cancer. (2014) 22:1089–96. doi: 10.1007/s00520-013-2049-3 24292095 PMC4929053

